# Gender-differentiated pathways from childhood trauma to self-injury: rumination as a mediator in depressed adolescents

**DOI:** 10.1186/s40359-025-03440-2

**Published:** 2025-09-26

**Authors:** Mailin Zhou, Shijian Wang, Doudou Zheng, Jingya Li, Ying Yang

**Affiliations:** 1 Medical Integration and Practice Center, Shandong University, Jinan, 250012 Shandong China; 2 Child and Adolescent Psycho-behavioral Medicine Center, Shandong Mental Health Center, Shandong University, Jinan, 250014 Shandong China

**Keywords:** Depressed adolescents, Childhood trauma subtypes, Non-suicidal self-injury, Gender differences, Rumination

## Abstract

**Background:**

Amid the global escalation of adolescent depression and non-suicidal self-injury (NSSI), childhood trauma has been identified as a critical risk factor, yet how specific trauma subtypes contribute to NSSI remains unclear. This study aims to explore the relationship between childhood trauma subtypes and NSSI in depressed adolescents, focusing gender differences, and verify the mediating role of rumination.

**Methods:**

This cross-sectional study included 2,006 clinically depressed adolescents (aged 10–19), who were recruited through convenience sampling from a psychiatric hospital in China (2024–2025). Validated scales (Childhood Trauma Questionnaire [CTQ], Ruminative Response Scale [RRS], Ottawa Self-Injury Inventory [OSI]) were administered. Binary logistic regression, subgroup analysis, and mediation analysis were used to examine the relationships between variables.

**Results:**

NSSI prevalence reached 69.3% in depressed adolescents (female: 77.6% vs. male: 52.1%). Emotional neglect (EN, odds ratio [OR] = 1.82), emotional abuse (EA, OR = 2.06), and physical abuse (PA, OR = 2.55) were associated with NSSI. Gender difference analysis revealed that females were more sensitive to EN (OR = 2.30) and EA (OR = 2.06), while males were more sensitive to EA (OR = 2.07) and PA (OR = 2.12). A single trauma subtype significantly increased the risk of NSSI in females, whereas males required at least two trauma subtypes to significantly increase NSSI risk. Rumination played a mediating role between all trauma subtypes and NSSI, with sexual abuse (SA) showing the highest mediation effect (78.02%), indicating a full mediation effect.

**Conclusions:**

The childhood trauma-NSSI association in depressed adolescents demonstrates gender difference, with rumination functioning as the central mechanistic pathway. Interventions must prioritize trauma subtype- and gender-tailored strategies to disrupt rumination pathways.

**Clinical trial number:**

Not applicable.

**Supplementary Information:**

The online version contains supplementary material available at 10.1186/s40359-025-03440-2.

## Introduction

Adolescent mental health has received growing attention, with depression being the leading cause of health-related disabilities and a major contributor to the global disease burden [1]. A meta-analysis reported a 24.3% prevalence of depression among adolescents in mainland China [2]. A systematic review of longitudinal studies found that nonsuicidal self-injury (NSSI) peaks in mid-adolescence (around ages 15–16), with depression as one of its strongest correlates [3]. Studies show that the incidence of NSSI among depressed adolescents is as high as 40%-60% [4]. Non-suicidal self-injury (NSSI) is associated with an increased risk of suicidal behavior, with cognitive factors (e.g., rumination) and exposure to painful and provocative events (e.g., childhood trauma) serving as key influences [5]. Beyond elevating suicide risk, NSSI aggravates psychiatric symptoms and functional impairments, constituting a significant global public health concern [6].

Exploring the triggering factors of NSSI is crucial for prevention and intervention. Nock’s integrated model emphasizes the role of early distal risk factors, particularly childhood trauma, in triggering NSSI behavior [7]. Childhood trauma mainly includes five dimensions: emotional neglect, emotional abuse, physical neglect, physical abuse, and sexual abuse [8]. Surveys indicate that approximately 65% of individuals who self-harm have experienced at least one form of childhood trauma, and about 50% have encountered multiple types of childhood trauma [9], with a dose-response relationship between the two [10]. Systematic reviews have shown that all types of childhood trauma are significantly associated with NSSI [11]. Moreover, adverse childhood experiences are highly indicative of suicidal behavior [12]. However, there is still insufficient understanding of the relationship between childhood trauma subtypes and NSSI in depressed adolescents, as well as the gender differences in this relationship and the dose-response relationship between them.

The mechanisms through which childhood trauma subtypes influence NSSI behaviors remain unclear. Recently, increasing attention has been paid to the role of cognitive factors in the development of psychological issues. Rumination, defined as a repetitive and passive focus on negative emotions and their causes and consequences, rather than active problem-solving [13]. Longitudinal evidence indicates that NSSI is bidirectionally linked with psychological distress and amplifies rumination, perpetuating a cycle of cognitive dysregulation in trauma-exposed adolescents [14]. Individuals with NSSI exhibit heightened negative affect, rumination, and self-criticism in daily life, with intensified interactions between emotional and cognitive states—highlighting affective-cognitive vulnerabilities following childhood trauma [15]. This suggests the presence of a maladaptive feedback loop in which trauma and behavioral processes interact in a reciprocal and self-sustaining manner over time, under the influence of rumination. For instance, rumination may not only mediate the effects of trauma on NSSI, but can also be intensified by the emotional consequences of self-injury, such as shame or intrusive thoughts. The Emotional Cascade Model (ECM) of NSSI [16] provides a conceptual framework for this recursive dynamic, positing that individuals engage in self-injury to escape the distress amplified by persistent rumination. Moreover, previous research demonstrates that adolescents with histories of trauma are more likely to develop ruminative thinking styles [17–19]. Although prior research has shown that rumination partially mediates the relationship between childhood trauma and NSSI, the mediating role of rumination in the link between specific trauma subtypes (e.g., emotional neglect, physical abuse) and NSSI remains insufficiently understood.

In summary, this study aims to explore the associations between childhood trauma subtypes and NSSI, the gender differences, and the mediating role of rumination in these relationships among depressed adolescents.

## Methods

### Participants and data collection

A convenience sampling method was employed to recruit adolescent patients diagnosed with depression from the outpatient and inpatient units of the child and adolescent psychiatry department at a mental health hospital in China as research participants. Inclusion criteria included: (1) Diagnosis of depression based on the Diagnostic and Statistical Manual of Mental Disorders, Fifth Edition (DSM-5) by two experienced psychiatrists. (2) Age between 10 and 19 years old. (3) Participants were able to understand the questionnaire content and complete it truthfully. (4)All participants and their legal guardians provided informed consent after being fully informed about the study, participated voluntarily, and retained the right to withdraw at any time. Exclusion criteria included: (1) Currently experiencing acute episodes of severe mental disorders (e.g., schizophrenia, bipolar disorder); (2) Severe substance abuse problems that may interfere with the evaluation of self-injury behaviors; (3) Neurodevelopmental disorders (e.g., intellectual disability, autism spectrum disorder, attention deficit hyperactivity disorder); (4) acute and transient psychotic disorders or dissociative (conversion) disorders (based on DSM-5) resulting from recent major traumatic events (e.g., the loss of a loved one) may hinder the completion of questionnaires.

Questionnaires were completed online via electronic QR codes, enhancing the convenience and transparency of the research process. Data collection was conducted in a quiet, private, and uniformly arranged room to minimize environmental interference and ensure participants’ privacy and focus. Researchers provided standardized instructions to participants to help them accurately understand the questionnaire content and task requirements. The completion process takes approximately 15–20 min. Data collection for this study occurred between June 2024 and February 2025, though the study is ongoing. This study was approved by the relevant institutional ethics committee (2024-R-156) and informed consent was obtained from all participants.

### Measures

Childhood trauma was assessed using the Childhood Trauma Questionnaire (CTQ) [20], which is designed to measure five subtypes of abuse and neglect in children or adolescents: emotional neglect, emotional abuse, physical neglect, physical abuse, and sexual abuse. The descriptions provided are conceptual definitions derived from the CTQ manual. To enhance clarity, representative item examples are included. Emotional neglect refers to the failure to meet essential emotional and psychological needs (e.g., “I felt loved” ; emotional abuse involves verbal assaults or behaviors that may humiliate or threaten the child (e.g., “People in my family said hurtful or insulting things to me”); physical neglect indicates inadequate provision of basic physical necessities (e.g., “There was someone to take me to the doctor if I needed it” ; physical abuse denotes deliberate physical harm (e.g., “I was punished with a belt, a board, a cord, or some other hard object”); and sexual abuse refers to any form of sexual contact or behavior imposed by an adult (e.g., “Someone tried to make me do sexual things or watch sexual things”). The questionnaire consists of 25 items rated on a 5-point Likert scale from 1 (never) to 5 (always), with higher scores indicating greater severity of abuse or neglect. The total score of the scale ranges from 25 to 125, with each of the five subscales ranging from 5 to 25; higher scores indicate more severe trauma. Cumulative childhood trauma (CCT) was created by summing the dichotomized scores of different subscales of childhood trauma, with a total score ranging from 0 to 5 (Cronbach’s α = 0.882). The Cronbach’s α coefficients for emotional neglect, emotional abuse, physical neglect, physical abuse, and sexual abuse were 0.842, 0.793, 0.504, 0.843, and 0.901, respectively.

Ruminative thinking was assessed using the 22-item Ruminative Response Scale (RRS) [21]. Although the RRS includes three subdimensions—symptom rumination, brooding, and reflective pondering—we used the total score to represent overall rumination severity in this study. The total score aligns with the Emotional Cascade Model and related theoretical frameworks that emphasize the cumulative role of rumination in emotional dysregulation. All items were rated on a 4-point Likert scale (1 = almost never to 4 = almost always), with higher scores indicating more severe rumination. The internal consistency of the total scale was excellent in this sample (Cronbach’s α = 0.956).

Participants were assessed for the presence of NSSI behavior using the Ottawa Self-Injury Inventory (OSI) [22]. They were asked to respond to the question: “In the past year, have you engaged in any of the following behaviors to intentionally harm yourself? These behaviors were not intended for suicide but may have resulted in bleeding, bruising, or pain”. Participants could respond with “Yes” or “No”, and those who answered “Yes” were classified as exhibiting NSSI behavior.

Adolescence is a key stage for balancing autonomy and caregiver connection. Nonsuicidal self-injury (NSSI), most common during this period, can strain family relationships. The NSSI Family Distress Cascade theory [23] posits that parents’ guilt, fear, and shame may prompt overcontrol, perceived by adolescents as intrusive, thereby worsening family functioning and sustaining NSSI. To account for contextual influences, the analysis included demographic covariates—age, gender, academic performance, personality type, parental marital status, parenting style, only-child status, socioeconomic status (SES), and place of residence. These factors may shape vulnerability to NSSI via different pathways, such as SES reflecting resource access and stress exposure, academic performance affecting self-esteem and peer relations, and parenting style influencing emotion regulation. In the current study, these covariates are examined in an exploratory manner to provide a more nuanced understanding of the trauma–NSSI relationship.

### Statistical analysis

Categorical variables were summarized using frequencies and proportions, while continuous variables were presented as means (standard deviations) to describe the participant characteristics. The logistic regression analysis was used to estimate the odds ratios (ORs) and 95% confidence intervals (CIs) for the association between childhood trauma subtypes and NSSI. Subgroup analysis was performed to examine whether there were gender differences in this association. Given the similar NSSI prevalence rates (all 85–88%) in the CCT = 3, CCT = 4, and CCT = 5 groups, these were combined into a single CCT ≥ 3 group, consistent with prior research practices [24, 25]. In Model 1, demographic characteristics were adjusted for, including gender, age, academic performance and personality. In Model 2, family environment was adjusted for, including parental marital status, parenting styles and only child. In Model 3, economic status was adjusted for, including family economic status, residence. In Model 4, suicidal ideation and rumination was adjusted. Finally, logistic regression-based mediation analysis was conducted to clarify the mediating role of rumination in the relationship between childhood trauma and NSSI in the fully adjusted model. Statistical analysis was performed using SPSS 27.0 and R 4.4.0.

### Exploratory analysis

Given the wide age range of 10–19 years, participants were categorized into three groups according to stages of adolescence: early (10–13 years), middle (14–16 years), and late adolescence (17–19 years). Additional stratified analyses were conducted to examine differences in the prevalence of depression and NSSI, as well as their associations, across these age stages.

## Results

This study included a total of 2,006 depressed adolescents, with 649 males (32.4%) and 1,357 females (67.6%). The results showed that the overall incidence rate of NSSI among depressed adolescents was 69.3%, with a total of 1,391 adolescents reporting NSSI. The incidence was 52.1% in males and 77.6% in females (χ² = 134.47, *P* < 0.001). Other detailed characteristics are shown in Table 1.

The results of the binary logistic regression are shown in Table 2. The subtype incidence rates of childhood trauma were as follows: emotional neglect 53.7%, emotional abuse 29.1%, physical neglect 49.1%, physical abuse 14.9%, and sexual abuse 9.8%. In Model 3, after controlling for demographic and economic factors, emotional neglect, emotional abuse, physical neglect, and physical abuse were identified as direct risk factors for adolescent NSSI behaviors. However, in Model 4, after controlling for rumination, physical neglect was no longer directly associated with NSSI. Additionally, the subtypes of childhood trauma exhibited a cumulative effect on NSSI behaviors, meaning that the more trauma subtypes experienced, the higher the risk of NSSI.

The gender differences are presented in Table 3. Although statistical analysis revealed no significant gender differences in the relationship between childhood trauma subtypes and NSSI, the data indicate that females experienced more childhood trauma than males. Males were primarily affected by emotional abuse and physical abuse, while females were more impacted by emotional neglect and emotional abuse. Females appear to be more sensitive to childhood trauma, with the experience of a single trauma subtype significantly increasing the risk of NSSI, whereas males needed to experience multiple trauma subtypes to significantly increase their risk.

**Fig. 1:**
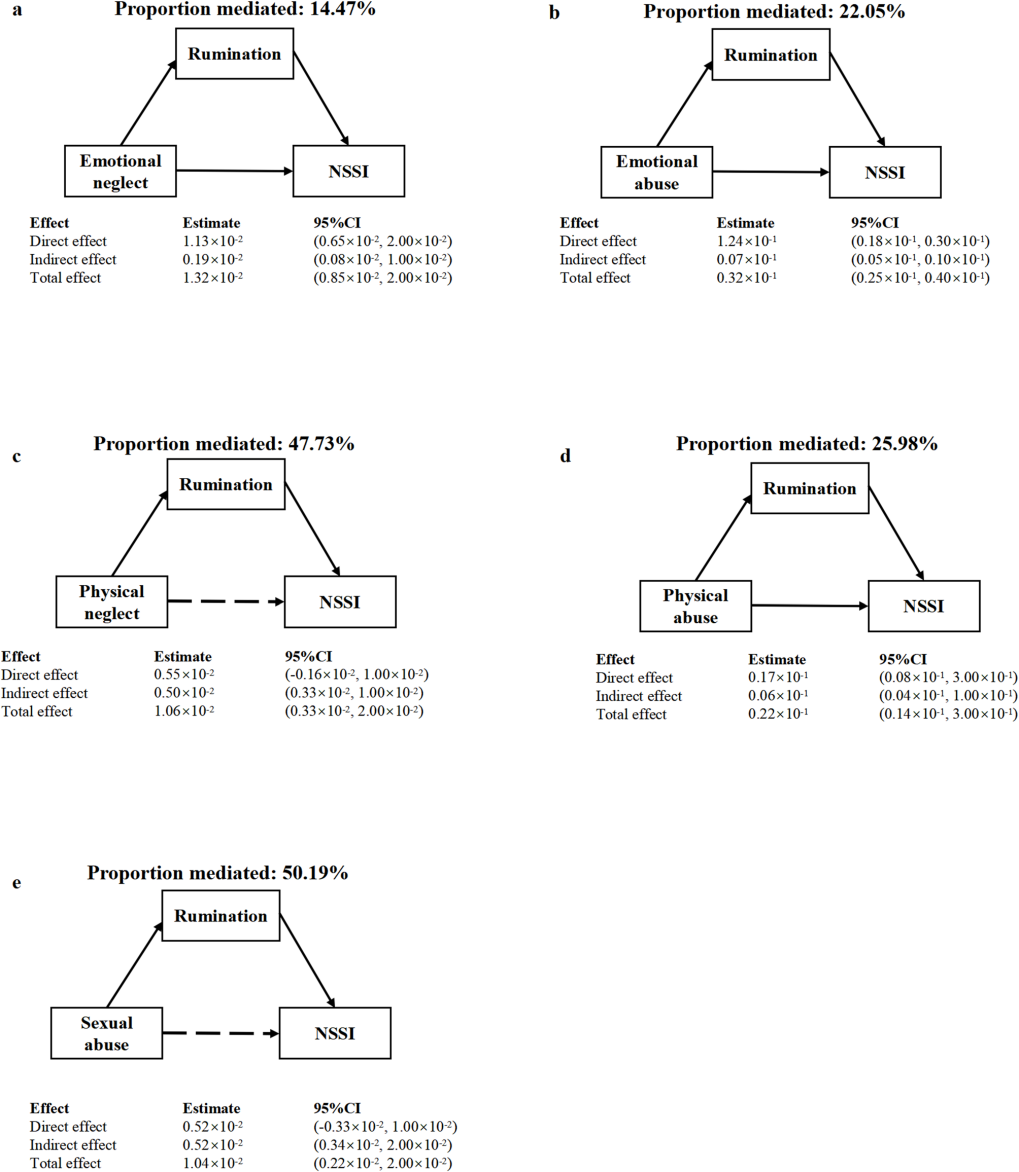
Mediating effect of Rumination between childhood trauma subtypes (**a-e**) and NSSI; The 95% *CI* of these estimates was computed using the bootstrap method (1,000 samples); The model was adjusted for gender, age, performance, personality, parental marriage, parenting styles, only child, residence and SES. Abbreviations: NSSI, non-suicidal self-injury

The mediation effect results are shown in Fig. 1. Five trauma subtypes—emotional neglect, emotional abuse, physical neglect, physical abuse, and sexual abuse—were found to influence NSSI through rumination as a mediating variable. The mediation proportions for emotional neglect, emotional abuse, physical neglect, physical abuse, and sexual abuse were 14.47%, 22.05%, 47.73%, 25.98%, and 50.19%, respectively. Overall, rumination played a significant mediating role in the relationship between different types of trauma subtypes and NSSI.

The results of the exploratory analyses are as follows: After stratifying by age, the results showed clear heterogeneity in the risk factors for NSSI across early adolescence (ages 10–13), middle adolescence (ages 14–16), and late adolescence (ages 17–19) (see Supplementary Table S1). In early adolescence, only emotional abuse significantly increased the risk of NSSI. In middle adolescence, the risk factors expanded to include emotional neglect, emotional abuse, and physical abuse. In late adolescence, emotional neglect, emotional abuse, and physical neglect all emerged as significant risk factors.

**Table 1:** Characteristics of participants (*n*, %)

Subgroup	Total	Male (*n* = 649)		Female (*n =* 1357)		χ^2^/t	*P*
		Non-NSSI (*n* = 311)	NSSI (*n* = 338)	Non-NSSI (*n* = 304)	NSSI (*n* = 1053)		
**Performance**						2.05	0.36
Upper Tier	348 (17.35)	70 (22.50)	68 (20.12)	45 (14.80)	165 (15.67)		
Middle Tier	972 (48.45)	142 (45.66)	150 (44.38)	160 (52.63)	520 (49.38)		
Lower Tier	686 (34.20)	99 (31.84)	120 (35.50)	99 (32.57)	368 (34.95)		
**Personality**						8.04^*^	0.02
Introverted	899 (44.82)	139 (44.70)	176 (52.07)	134 (44.08)	450 (42.74)		
Extroverted	243 (12.11)	47 (15.11)	30 (8.88)	46 (15.13)	120 (11.40)		
Neutral	864 (43.07)	125 (40.19)	132 (39.05)	124 (40.79)	483 (45.86)		
**Parents’ marital status**						2.32	0.51
First marriage	1710 (85.24)	268 (86.17)	297 (87.87)	266 (87.50)	879 (83.48)		
Divorced	147 (7.33)	27 (8.68)	16 (4.73)	16 (5.26)	88 (8.36)		
One deceased	33 (1.65)	4 (1.29)	6 (1.78)	5 (1.64)	18 (1.71)		
Remarried	116 (5.78)	12 (3.86)	19 (5.62)	17 (5.60)	68 (6.45)		
**Only child**						0.01	0.94
Yes	573 (28.56)	93 (29.90)	121 (35.80)	82 (26.97)	277 (26.31)		
No	1433 (71.44)	218 (70.10)	217 (64.20)	222 (73.03)	776 (73.69)		
**Parenting styles**						46.55^***^	< 0.001
Authoritarian	321 (16.00)	52 (16.72)	66 (19.53)	44 (14.47)	159 (15.10)		
Neglectful	287 (14.31)	27 (8.68)	47 (13.91)	28 (9.21)	185 (17.57)		
Authoritative	461 (22.98)	46 (14.79)	80 (23.67)	67 (22.04)	268 (25.45)		
Permissive	144 (7.18)	31 (9.97)	26 (7.69)	24 (7.89)	63 (5.97)		
Democratic	793 (39.53)	155 (49.84)	119 (35.20)	141 (46.38)	378 (35.90)		
**Residence**						6.34^*^	0.04
Urban	1050 (52.34)	163 (52.41)	167 (49.41)	161 (52.96)	559 (53.09)		
Town	690 (34.40)	91 (29.26)	121 (35.80)	103 (33.88)	375 (35.61)		
Rural	266 (13.26)	57 (18.33)	50 (14.79)	40 (13.16)	119 (11.30)		
**SES**						27.11^***^	< 0.001
High	56 (2.80)	16 (5.14)	10 (2.96)	14 (4.61)	16 (1.52)		
Upper-middle	740 (36.89)	127 (40.84)	112 (33.14)	129 (42.43)	372 (35.33)		
Lower-middle	1089 (54.29)	151 (48.55)	182 (53.85)	150 (49.34)	606 (57.55)		
Low	121 (6.02)	17 (5.47)	34 (10.05)	11 (3.62)	59 (5.60)		
**Adolescence**						13.7^***^	< 0.001
Early adolescence	445 (22.18)	72 (23.15)	40 (11.83)	77 (25.33)	256 (24.31)		
Middle adolescence	1024 (51.05)	158 (50.80)	190 (56.21)	123 (40.46)	553 (52.52)		
Late adolescence	537 (26.77)	81 (26.05)	108 (31.96)	104 (34.21)	244 (23.17)		
**CCT**						27.90^***^	< 0.001
0	565 (28.17)	140 (45.02)	87 (25.74)	130 (42.76)	208 (19.75)		
1	475 (23.68)	75 (24.12)	81 (23.96)	75 (24.67)	244 (23.17)		
2	502 (25.02)	66 (21.22)	82 (24.26)	69 (22.70)	285 (27.07)		
≥ 3	464 (23.13)	30 (9.64)	88 (26.04)	30 (9.87)	316 (30.01)		
**NSSI Frequency (6 months)**						40.71^***^	< 0.001
Never	208 (14.95)	-	79 (23.37)	-	129 (12.25)		
1–5 times	583 (41.91)	-	156 (46.15)	-	427 (40.55)		
Monthly	167 (12.01)	-	26 (7.69)	-	141 (13.39)		
Weekly	301 (21.64)	-	57 (16.86)	-	244 (23.17)		
Daily	132 (9.49)	-	20 (5.93)	-	112 (10.64)		
**Suicide ideation**						98.80^***^	< 0.001
No	426 (21.24)	175 (56.27)	48 (14.20)	115 (37.83)	88 (8.36)		
Yes	1580 (78.76)	136 (43.73)	290 (85.80)	189 (62.17)	965 (91.64)		
**Age**,** M (SD)**	15.15 (2.05)	15.15 (2.20)	15.55 (1.82)	15.27 (2.39)	14.99 (1.95)	3.14^**^	< 0.01
**Age of onset of NSSI M (SD)**	12.97 ()1.95	-	13.46 (2.13)	-	12.81 (1.87)	5.10^***^	< 0.001
**Rumination**,** M (SD)**	57.07 (16.64)	45.82 (16.08)	58.99 (14.07)	48.94 (16.20)	62.12 (15.15)	-8.31^***^	< 0.001

Abbreviations: CCT, cumulative childhood trauma; M, mean; NSSI, non - suicidal self - Injury; SES, socioeconomic status; SD, standard deviation; ^*^P < 0.05, ^**^P < 0.01, ^***^P < 0.001

**Table 2:** Relationships between childhood trauma subtypes and NSSI

Group	*n* (%)	OR (95%)				
		Unadjusted	Model1^a^	Model2^b^	Model3^c^	Model4^d^
**Emotional Neglect**						
No	929 (46.31)	1.00 [Reference]	1.00 [Reference]	1.00 [Reference]	1.00 [Reference]	1.00 [Reference]
**Yes**	1077 (53.69)	2.63 (2.16–3.20)^***^	2.38 (1.94–2.91)^***^	2.13 (1.71–2.64)^***^	2.06 (1.66–2.56)^***^	1.66 (1.31–2.11)^***^
**Emotional Abuse**						
No	1422 (70.89)	1[Reference]	1[Reference]	1[Reference]	1[Reference]	1[Reference]
Yes	584 (29.11)	4.39 (3.36–5.75)^***^	3.96 (3.01–5.21)^***^	3.55 (2.67–4.72)^***^	3.48 (2.62–4.64)^***^	2.07 (1.52–2.83)^***^
**Physical Neglect**						
No	1021 (50.90)	1.00 [Reference]	1.00 [Reference]	1.00 [Reference]	1.00 [Reference]	1.00 [Reference]
Yes	985 (49.10)	1.79 (1.47–2.17)^***^	1.76 (1.43–2.15)^***^	1.58 (1.28–1.95)^***^	1.55 (1.25–1.92)^***^	1.21 (0.96–1.53)
**Physical Abuse**						
No	1707 (85.09)	1.00 [Reference]	1.00 [Reference]	1.00 [Reference]	1.00 [Reference]	1.00 [Reference]
Yes	299 (14.91)	2.46 (1.79–3.39)^***^	2.92 (2.09–4.08)^***^	2.55 (1.81–3.58)^***^	2.50 (1.78–3.52)^***^	2.05 (1.41–2.98)^***^
**Sexual Abuse**						
No	1810 (90.23)	1.00 [Reference]	1.00 [Reference]	1.00 [Reference]	1.00 [Reference]	1.00 [Reference]
Yes	196 (9.77)	1.50 (1.06–2.11)^*^	1.48 (1.04–2.11)^*^	1.37 (0.95–1.97)	1.34 (0.93–1.94)	1.14 (0.76–1.70)
**CCT**						
0	565 (28.17)	1.00 [Reference]	1.00 [Reference]	1.00 [Reference]	1.00 [Reference]	1.00 [Reference]
1	475 (23.68)	1.98 (1.54–2.56)^***^	1.92 (1.48–2.51)^***^	1.87 (1.43–2.44)^***^	1.83 (1.40–2.40)^***^	1.61 (1.20–2.17)^**^
2	502 (25.02)	2.49 (1.92–3.22)^***^	2.34 (1.79–3.07)^***^	2.18 (1.65–2.89)^***^	2.12 (1.60–2.82)^***^	1.60 (1.17–2.19)^**^
≥ 3	464 (23.13)	6.16 (4.49–8.47)^***^	5.78 (4.16–8.02)^***^	5.16 (3.65–7.31)^***^	4.98 (3.51–7.06)^***^	2.82 (1.93–4.11)^***^

Abbreviations: CCT, cumulative childhood trauma; CI, confidence interval; NSSI, non - suicidal self - injury; OR, odds ratio

^a^Model 1: Adjusted for gender, age, performance and personality

^b^Model 2: Adjusted for gender, age, performance, personality, parental marriage, parenting styles and only child

^c^Model 3: Adjusted for gender, age, performance, personality, parental marriage, parenting styles, only child, residence and SES

^d^Model 4: Adjusted for gender, age, performance, personality, parental marriage, parenting styles, only child, residence SES and suicide ideation, rumination

**Table 3:** Relationships between childhood trauma subtypes and NSSI by gender

Group	Male (*n* = 649)			Female (*n* = 1357)			ROR^c^	*P*
	*n* (%)	OR (95% CI)^a^	aOR (95% CI)^b^	*n* (%)	OR (95% CI) ^a^	aOR (95% CI) ^b^		
**Emotional Neglect**								
No	364 (56.09)	1.00 [Reference]	1.00 [Reference]	565 (41.64)	1.00 [Reference]	1.00 [Reference]	0.57	0.06
Yes	285 (43.91)	1.73 (1.26–2.36)^***^	1.21 (0.83–1.78)	792 (58.36)	3.01 (2.31–3.93)^***^	2.13 (1.55–2.93)^***^		
**Emotional Abuse**								
No	518 (79.82)	1.00 [Reference]	1.00 [Reference]	904 (66.62)	1.00 [Reference]	1.00 [Reference]	1.10	0.48
Yes	131 (20.18)	4.43 (2.82–6.96)^***^	2.21 (1.30–3.76)^**^	453 (33.38)	3.75 (2.66–5.29)^***^	2.01 (1.36–2.96)^***^		
**Physical Neglect**								
No	348 (53.62)	1.00 [Reference]	1.00 [Reference]	673 (49.59)	1.00 [Reference]	1.00 [Reference]	0.95	0.90
Yes	301 (46.38)	1.70 (1.25–2.32)^***^	1.18 (0.80–1.73)	684 (50.41)	1.83 (1.41–2.38)^***^	1.24 (0.91–1.68)		
**Physical Abuse**								
No	535 (82.43)	1.00 [Reference]	1.00 [Reference]	1172 (86.37)	1.00 [Reference]	1.00 [Reference]	1.41	0.30
Yes	114 (17.57)	3.10 (1.98–4.86)^***^	2.32 (1.36–3.96)^***^	185 (13.63)	2.64 (1.63–4.28)^***^	1.65 (0.98–2.78)		
**Sexual Abuse**								
No	591 (91.06)	1.00 [Reference]	1.00 [Reference]	1219 (89.83)	1.00 [Reference]	1.00 [Reference]	1.11	0.65
Yes	58 (8.94)	1.70 (0.97–2.97)	1.19 (0.61–2.31)	138 (10.17)	1.34 (0.85–2.11)	1.07 (0.64–1.78)		
**CCT**								
0	227 (34.98)	1.00 [Reference]	1.00 [Reference]	338 (24.91)	1.00 [Reference]	1.00 [Reference]		
1	156 (24.04)	1.74 (1.15–2.63)^**^	1.46 (0.90–2.39)	319 (23.51)	2.03 (1.45–2.85)^***^	1.69 (1.16–2.46)^**^	0.87	0.93
2	148 (22.80)	2.00 (1.31–3.04)^*^	1.57 (0.94–2.60)	354 (26.09)	2.58 (1.83–3.64)^***^	1.68 (1.12–2.51)^*^	0.93	0.98
≥ 3	118 (18.18)	4.72 (2.88–7.73)^***^	2.18 (1.21–3.93)*	346 (25.49)	6.58 (4.27–10.16)^***^	3.34 (2.01–5.54)^***^	0.65	0.57

Abbreviations: CCT, cumulative childhood trauma; CI, confidence interval; OR, odds ratio; ^*^P < 0.05, ^**^P < 0.01, ^***^P < 0.001; ROR, relative odds ratio

a: Unadjusted model

b: Adjusted for gender, age, performance, personality, parental marriage, parenting styles, only child, residence SES and suicide ideation, rumination

c: Calculated by adjusted OR

## Discussion

The present study advances current understanding of NSSI etiology by exploring potential gender-specific patterns and identifying rumination as a pivotal mediator across all childhood trauma subtypes—a dual contribution that addresses critical gaps in prior research. While existing literature broadly links childhood trauma to NSSI, our findings suggest a possible “low-threshold, high-sensitivity” response among females, where exposure to a single trauma subtype may increase risk, whereas males may require cumulative trauma exposure (≥ 2 subtypes) to manifest comparable risk. Although this pattern did not reach statistical significance in gender-by-trauma interaction tests, it may represent a promising hypothesis for future research. Developmentally, females enter puberty earlier than males, accelerating cortical-limbic circuit maturation during early adolescence. This heightens sensitivity to social-evaluative threats (e.g., emotional neglect/abuse), particularly as the female brain exhibits amplified limbic (amygdala) reactivity to interpersonal stressors compared to males [26, 27]. This threshold divergence challenges homogeneous trauma-NSSI models and underscores the necessity of gender-tailored prevention frameworks. In practice, such frameworks may involve trauma-informed psychoeducation and coping skills training tailored to gender-specific trauma sensitivities. For females, priorities include addressing emotional neglect and abuse, enhancing social support, and reducing rumination; for males, emphasis may be placed on managing physical abuse, reducing self-blame, and fostering healthy emotional expression. Furthermore, this study validates the core cognitive mediating role of rumination in the relationship between childhood trauma and NSSI, establishing it as a novel transdiagnostic therapeutic target for intervention development.

Among all childhood trauma subtypes, all except sexual abuse were directly associated with NSSI. However, some studies suggest that sexual abuse is also an important trigger for NSSI [28]. One possible explanation is that, within the Chinese cultural context, the stigma surrounding sexual victimization may lead adolescents to conceal such experiences in research, even in confidential settings. The reliance on self-report questionnaires further increases susceptibility to social desirability bias, whereby experiences perceived as shameful may be minimized or omitted. Moreover, the relatively low observed prevalence of sexual abuse in our sample—potentially influenced by sociocultural norms and reporting practices—may limit statistical power to detect direct effects. These factors, together with prevailing cultural values emphasizing female chastity and male masculinity, may jointly obscure the relationship between sexual abuse and NSSI in this population [29].

Although the statistical significance was not reached, gender-specific risk patterns have important clinical implications. First, emotional abuse is a common risk factor for NSSI behavior in both males and females, which is consistent with previous research [30]. Emotional abuse refers to the long-term negative attitudes of caregivers toward children, involving verbal attacks on a person’s sense of worth or well-being, including criticism, threats, humiliation, blaming, and insults [31]. Unlike other forms of abuse, many people suffer from long-term emotional abuse without being aware of it [32]. Emotional abuse is one of the most damaging forms of abuse [33], causing lasting harm to a child’s self-esteem and hindering the development of effective emotional regulation skills [34]. These adolescents may exhibit reduced HPA axis response levels [35], making them less sensitive to pain and engaging in NSSI as a coping mechanism to regulate distress [36].

Additionally, physical abuse is an independent risk factor for NSSI in boys, which also confirms previous research findings [37]. In traditional family concepts and educational models, some parents adhere to the outdated idea that “a filial child is raised with a stick” and believe that children need to be corrected through severe corporal punishment to shape their character [38]. Social role theory suggests [39] that societal expectations place a greater social responsibility on boys in adulthood, which encourages parents to adopt stricter parenting strategies for boys than for girls. This belief leads to the possibility that boys may experience physical punishment more frequently than girls during their upbringing. Frequent corporal punishment, as a direct form of physical abuse, severely undermines boys’ self-esteem, causing them to doubt their own worth [37]. Self-blame may explain the association between physical abuse and NSSI. Children may learn to blame themselves for the physical abuse they endure from their parents, especially if they had misbehaved before the incident [37]. NSSI, as a form of self-punishment, is used to alleviate the tension and emotional pain arising from constant self-blame and parental criticism.

Emotional neglect is a distinct risk factor for NSSI behavior in females. Research shows that emotional neglect can intensify an individual’s feelings of self-shame and negative self-beliefs, as this neglect implies that the individual is unworthy of others’ care and attention [40]. If a person grows up without caregivers who provide emotional support, security, and are sensitive enough to respond to the child’s needs, it may hinder the development of healthy interpersonal relationships or close friendships [41]. This is particularly true for females, who typically have higher emotional needs and more delicate emotional expressions. Emotional neglect may lead them to feel emotionally isolated, prompting them to engage in NSSI behaviors as a way to elicit care, support, and recognition from others.

This study found that female adolescents with depression experience a greater variety of trauma types, showing a “low-threshold, high sensitivity” pattern between childhood trauma exposure and the risk of NSSI. This suggests the existence of gender-specific stress response mechanisms. In addition to the higher sensitivity of females to trauma, another possible explanation is that males, after experiencing trauma, may exhibit more outward behaviors (e.g., violence or aggression) [26], which could influence the onset of NSSI. Furthermore, the societal expectation for males to be resilient and strong may act as a psychological buffer when they encounter trauma. However, when the intensity of trauma exceeds the threshold of this buffering mechanism, it may lead to more severe consequences [42].

The mediating role of rumination in NSSI behavior was observed across all five trauma subtypes, but the strength of the effect varied significantly. Although sexual abuse showed a high mediating proportion (78.02%) with complete mediation, the small number of reported cases and potential underreporting due to cultural stigma limit the robustness of this finding. Thus, this result should be interpreted cautiously and confirmed in larger, more diverse samples. This suggests that the core mechanism triggering NSSI in sexual abuse victims lies in the intrusive rumination triggered by traumatic memories (such as repeatedly reliving the victimization scene) [43]. This aligns closely with the unique shame and low self-worth typically associated with sexual trauma [44]. Victims of sexual abuse often become trapped in a “secret-rumination” cycle due to social stigmatization: the unspeakable nature of the traumatic experience leads to repetitive self-reflection [45, 46], and the intensified self-blame resulting from rumination further exacerbates the impulse for NSSI.

This study found that rumination plays a significant mediating role between both physical and emotional neglect and NSSI. Compared to other forms of abuse, neglect has a unique impact on children’s cognitive development [47]. Neglect hinders the establishment of close and stable emotional connections between individuals and their caregivers, as the lack of psychological guidance from caregivers deprives infants of their understanding of their inner world and pain in ways that are distinct from abuse. This disruption affects emotional regulation and the subsequent development of interpersonal functioning [48]. Neglect is particularly prevalent among left-behind children [49]. Due to prolonged absence of warmth and support from their families, these children often ruminate on these unpleasant experiences. According to the emotional cascade model, NSSI can serve as a way to divert attention from rumination, with individuals attempting to alleviate the pain and distress caused by rumination through this extreme behavior [16]. Consistent with cognitive–affective models of NSSI, recent longitudinal research demonstrates that NSSI, rumination, and psychological distress can be mutually reinforcing over time, forming a maladaptive cycle in which NSSI not only results from heightened rumination but also exacerbates it [14]. This bidirectional relationship suggests that interventions targeting rumination may help disrupt this cycle, thereby reducing both the onset and maintenance of NSSI.

Rumination also plays a partial mediating role between both physical and emotional abuse and NSSI. In abusive situations, the instillation of contradictory emotions, such as the justification “hitting you is for your own good”, leads adolescents into a state of cognitive confusion between love and hate. This polarity reversal causes complex and conflicting psychological pain in adolescents. In such a cultural environment, they are unable to express their inner emotions in a healthy way and can only resort to NSSI behaviors to communicate their inner pain and struggles [50].

After stratifying by age, the results showed clear heterogeneity in the risk factors for NSSI across different stages of adolescence. In early adolescence, the prefrontal cortex is still undergoing rapid development, while the amygdala responds more strongly to threatening stimuli [51]. Emotional abuse—such as criticism, humiliation, or threats—can directly disrupt the formation of self-worth and emotional regulation circuits during this stage, leading children to form a pervasive negative view of themselves [40]. When emotional regulation skills are still immature, emotional abuse can become an overwhelming source of stress, prompting them to use NSSI to relieve the heightened arousal and distress triggered by ongoing psychological attacks [52]. Middle adolescence is a period of rapid growth in peer relationships, self-identity, and independence [53, 54]. Emotional neglect means a lack of support from parents or primary caregivers, depriving adolescents of a critical emotional safety net during a key stage of self-construction. Emotional abuse further erodes self-esteem and the sense of belonging [55]; Physical abuse at this stage is often perceived as an extreme signal of control or rejection within relationships, amplifying feelings of social exclusion and self-denial [56]. The combined effect of these three types of trauma may significantly raise the likelihood of NSSI by increasing the cumulative load of shame, isolation, and self-aggressive thoughts.n late adolescence, young people gain more autonomy, and parental control may shift from direct punishment to more distant forms of interaction [57]. At this stage, the lack of emotional support, together with ongoing emotional devaluation, can undermine self-efficacy and hope for the future [58]; Insufficient practical care—such as in diet, health management, or daily needs—can also be interpreted as emotional coldness and a lack of responsibility, deepening feelings of isolation and neglect. These experiences may drive adolescents to use NSSI as a way to regulate emotions or express distress [59, 60]. Moreover, the impact of cumulative childhood trauma (CCT) on NSSI becomes more pronounced with age, possibly because multiple types of trauma accumulate and solidify over time [61]. Early traumatic experiences, through repeated recall and meaning-making, may gradually be internalized into stable negative self-schemas. These, in turn, can heighten emotional reactivity via neural sensitization, increasing vulnerability to self-injurious behavior in later developmental stages [51, 62].

In addition, this study found that gender differences in the impact of emotional abuse on NSSI emerged only during middle adolescence, with the effect being more pronounced among boys. This stage is a critical period for the rapid formation of male self-identity and awareness of social roles, during which both society and families tend to place greater expectations on boys to be “strong” and “independent” [63]. At this time, emotional abuse not only directly undermines self-esteem but also clashes with gender role expectations. On the one hand, boys are expected to suppress vulnerable emotions and are less likely to seek help from others [63]; On the other hand, their neural systems for emotional regulation and impulse control are not yet fully mature, making it easier for negative emotions to accumulate [64]. Lacking both emotional support channels and socially accepted ways to express vulnerability, such emotions are often released through aggression or self-injury. In this context, NSSI may serve as a covert outlet for emotional distress [65].

### Strengths and limitations

This study has several notable strengths. It targets adolescents with depression, a high-risk group, and uses a large sample size, ensuring the findings are both significant and relevant for improving mental health in this population. The study carefully categorizes childhood trauma subtypes and examines their unique effects, controlling for important covariates such as age, gender, and family environment. This approach enhances the internal validity of the findings, providing more accurate insights into the relationships between these variables. Additionally, the research highlights gender differences in the link between childhood trauma and NSSI, offering valuable evidence for gender-specific interventions. Despite these strengths, the study has some limitations. The use of convenience sampling introduces selection bias, which restricts the generalizability of the findings. Additionally, the reliance on self-reported data from questionnaires may lead to recall bias and social desirability bias, potentially affecting the accuracy of the data. Additionally, due to the cross-sectional nature of the study, we cannot rule out the possibility of reverse causality. For example, engagement in NSSI may heighten subsequent rumination or influence the recall and reporting of childhood trauma. In our study, NSSI was assessed as a dichotomous variable based on self-reported engagement in the past year. This approach conflates individuals who engaged in NSSI only once with those who self-injure frequently. Given that individuals with NSSI addiction may exhibit different ruminative patterns compared to non-addicted counterparts, this simplification may obscure important clinical distinctions. We agree that future research should incorporate frequency-based measures of NSSI to better capture the complexity and heterogeneity of self-injurious behavior. Furthermore, given the psychiatric setting, it is possible that some adolescents were experiencing ongoing trauma at the time of assessment. This overlap between ongoing and past trauma may have intensified rumination and increased the likelihood of NSSI, thereby potentially amplifying the observed associations. Because our measures did not distinguish between ongoing and past trauma, the results should be interpreted as reflecting cumulative trauma exposure. Future longitudinal studies are needed to disentangle their temporal and interactive effects. Moreover, the gender imbalance in NSSI prevalence—higher in females than males—may affect the stability of male subgroup analyses. Although the male sample size (*n* = 649) was adequate, future studies should aim for more balanced gender representation to improve the reliability of gender-specific findings.

## Conclusions

This study examines the association between childhood trauma subtypes and NSSI in depressed adolescents. Gender analyses reveal that females are more affected by emotional neglect and abuse, while males are more sensitive to emotional and physical abuse. The number of trauma subtypes shows a dose-response relationship with NSSI: females exposed to a single trauma subtype significantly increase their risk of NSSI, whereas males need to accumulate at least two subtypes to manifest a risk. Rumination mediates this relationship, particularly in sexual abuse, where it fully mediates the effect. Future research should focus on gender-specific interventions targeting trauma exposure and rumination to reduce NSSI risk.

## Supplementary Information

Below is the link to the electronic supplementary material.

Supplementary Material 1

## Data Availability

Due to participant privacy protection, the data cannot be publicly shared. To access the data, please contact the corresponding author.

## Abbreviations

NSSI: Non-suicidal self-injury
CI: Confidence interval
ORs: The odds ratios
ECM: The emotional cascade model
CCT: Cumulative childhood trauma
CTQ: Childhood trauma questionnaire
RRS: Ruminative response scale
