# Supplementary material for: Gender-differentiated pathways from childhood trauma to self-injury: rumination as a mediator in depressed adolescents
Source: BMC Psychol. 2025 Sep 26;13:1057. doi: 10.1186/s40359-025-03440-2 (PMC12465670; doi:10.1186/s40359-025-03440-2)
Supplement: Supplementary file 1 — Supplementary Material 1 [file 40359_2025_3440_MOESM1_ESM.docx]

**Table S1. Effects of Childhood Trauma on Non-Suicidal Self-Injury Across Different Stages of Adolescence**

| **Group** | **Early adolescence** | | | **Middle adolescence** | | | | **Late adolescence** | | |
| --- | --- | --- | --- | --- | --- | --- | --- | --- | --- | --- |
|  | ***n*** | **OR (95%CI)** | ***P*** | ***n*** | **OR (95%CI)** | ***P*** | ***n*** | | **OR (95%CI)** | ***P*** |
| **Emotional Neglect** |  |  | 0.85 |  |  | 0.01 |  | |  | 0.003 |
| No | 190 (42.70) | 1[Reference] |  | 446 (43.55) | 1[Reference] |  | 293 (54.56) | | 1[Reference] |  |
| **Yes** | 255 (57.30) | 1.06 (0.59-1.89) |  | 578 (56.45) | 1.58 (1.11-2.25) |  | 244 (45.44) | | 2.00 (1.27-3.14) |  |
| **Emotional Abuse** |  |  | 0.03 |  |  |  |  | |  | 0.01 |
| No | 305 (68.54) | 1[Reference] |  | 730 (71.29) | 1[Reference] | 0.01 | 387 (72.07) | | 1[Reference] |  |
| Yes | 140 (31.46) | 2.23 (1.10-4.55) |  | 294 (28.71) | 1.94 (2.20-3.13) |  | 150 (27.93) | | 2.07 (2.21-3.56) |  |
| **Physical Neglect** |  |  | 0.38 |  |  | 0.55 |  | |  | 0.04 |
| No | 199 (44.72) | 1[Reference] |  | 505 (49.31) | 1[Reference] |  | 317 (59.03) | | 1[Reference] |  |
| Yes | 246 (55.28) | 0.78 (0.45-1.35) |  | 519 (50.69) | 1.11 (0.78-1.58) |  | 220 (40.97) | | 1.62 (1.04-2.53) |  |
| **Physical Abuse** |  |  | 0.32 |  |  | <0.001 |  | |  | 0.36 |
| No | 381 (85.62) | 1[Reference] |  | 870 (84.96) | 1[Reference] |  | 456 (84.92) | | 1[Reference] |  |
| Yes | 64 (14.38) | 1.53 (0.66-3.51) |  | 154 (15.04) | 3.51 (1.88-6.56) |  | 81 (15.08) | | 1.33 (0.72-2.47) |  |
| **Sexual Abuse** |  |  | 0.9 |  |  | 0.67 |  | |  | 0.36 |
| No | 390 (87.64) | 1[Reference] |  | 939 (91.70) | 1[Reference] |  | 481 (89.57) | | 1[Reference] |  |
| Yes | 55 (12.36) | 1.06 (0.46-2.43) |  | 85 (8.30) | 1.16 (0.60-2.25) |  | 56 (10.43) | | 1.39 (0.69-2.79) |  |
| **CCT** |  |  |  |  |  |  |  | |  |  |
| 0 | 110 (24.72) | 1[Reference] |  | 264 (25.78) | 1[Reference] |  | 191 (35.57) | | 1[Reference] |  |
| 1 | 99 (22.25) | 0.84 (0.40-1.73) | 0.63 | 253 (24.71) | 1.54 (0.99-2.38) | 0.06 | 123 (22.91) | | 1.98 (1.16-3.40) | 0.01 |
| 2 | 113 (25.39) | 0.51 (0.24-1.08) | 0.08 | 277 (27.05) | 1.48 (0.93-2.35) | 0.1 | 112 (20.86) | | 3.23 (1.74-5.98) | <0.001 |
| ≥3 | 123 (27.64) | 1.36 (0.56-3.30) | 0.5 | 230 (22.46) | 3.11 (1.70-5.68) | <0.001 | 111 (20.66) | | 2.94 (1.52-5.68) | 0.01 |

Note: CCT, cumulative childhood trauma; CI, confidence interval; OR, odds ratio.

Adjusted for gender, age, performance, personality, parental marriage, parenting styles, only child, residence SES and suicide ideation, ruminati

**Table S2. Effects of the Interaction Between Childhood Trauma and Gender on Non-Suicidal Self-Injury Across Different Stages of Adolescence**

|  | **Early adolescence** | | **Middle adolescence** | | **Late adolescence** | |
| --- | --- | --- | --- | --- | --- | --- |
|  | **OR (95% CI)^a^** | **P** | **OR (95% CI) ^a^** | **P** | **aOR (95% CI) ^b^** | **P** |
|  |  |  |  |  |  |  |
| **Emotional Neglect [ref No]** | 1.07 (0.41-2.77) | 0.89 | 1.25 (0.75-2.09) | 0.38 | 1.61 (0.82-3.19) | 0.17 |
| **Emotional Neglect * gender** | 0.99 (0.32-3.04) | 0.98 | 1.53 (0.78-2.99) | 0.22 | 1.42 (0.61-3.27) | 0.41 |
| **Emotional Abuse [ref No]** | 1.06 (0.31-3.63) | 0.93 | 4.43 (2.82-6.96) | <0.001 | 1.79 (0.78-4.11) | 0.17 |
| **Emotional Abuse * gender** | 4.43 (2.82-6.96) | 0.16 | 0.32 (0.12-0.84) | 0.02 | 1.27 (0.45-3.60) | 0.65 |
| **Physical Neglect [ref No]** | 0.53 (0.21-1.39) | 0.20 | 1.40 (0.84-2.35) | 0.20 | 1.25 (0.64-2.45) | 0.52 |
| **Physical Neglect * gender** | 1.73 (0.56-5.31) | 0.34 | 0.66 (0.34-1.30) | 0.23 | 1.55 (0.66-3.61) | 0.32 |
| **Physical Abuse [ref No]** | 1.17 (0.34-3.99) | 0.81 | 4.45 (1.98-10.01) | <0.001 | 1.60 (0.65-3.97) | 0.31 |
| **Physical Abuse * gender** | 1.66 (0.30-9.24) | 0.33 | 0.56 (0.17-1.86) | 0.34 | 0.72 (0.22-2.39) | 0.59 |
| **Sexual Abuse [ref No]** | 1.16 (0.32-4.22) | 0.82 | 2.50 (0.87-7.23) | 0.09 | 0.94 (0.32-2.81) | 0.91 |
| **Sexual Abuse * gender** | 0.85 (0.17-4.32) | 0.85 | 0.27 (0.07-1.02) | 0.05 | 2.39 (0.59-9.58) | 0.22 |
| **CCT[ref CCT=0]** |  |  |  |  |  |  |
| CCT = 1 | 1.19 (0.34-4.14)^**^ | 0.79 | 1.59 (0.83-3.04) | 0.16 | 1.85 (0.76-4.52) | 0.18 |
| CCT = 2 | 0.98 (0.27-3.56)^**^ | 0.97 | 1.76 (0.89-3.48) | 0.11 | 1.83 (0.75-4.50) | 0.19 |
| CCT ≥3 | 0.61 (0.15-2.50)^*^ | 0.49 | 4.41 (1.83-10.62) | <0.001 | 2.21 (0.84-5.82)^*^ | 0.11 |
| **CCT * gender** |  |  |  |  |  |  |
| **CCT = 1 * gender** | 0.59 (1.13-2.70) | 0.49 | 0.92 (0.39-2.20) | 0.86 | 1.12 (0.38-3.34) | 0.84 |
| **CCT = 2 * gender** | 0.43 (0.10-1.94) | 0.27 | 0.74 (0.30-1.79) | 0.50 | 2.73 (0.85-8.79) | 0.09 |
| **CCT =3 * gender** | 3.66 (0.63-21.45) | 0.15 | 0.52 (0.17-1.61) | 0.26 | 1.58 (0.47-5.29) | 0.46 |

Note: CCT, cumulative childhood trauma; CI, confidence interval; OR, odds ratio.

Adjusted for gender, age, performance, personality, parental marriage, parenting styles, only child, residence SES and suicide ideation, rumination
